# Mindfulness-Based Stress Reduction (MBSR) or Psychoeducation for the Reduction of Menopausal Symptoms: A Randomized, Controlled Clinical Trial

**DOI:** 10.1038/s41598-018-24945-4

**Published:** 2018-04-26

**Authors:** Carmen Wong, Benjamin Hon-Kei Yip, Ting Gao, Kitty Yu Yuk Lam, Doris Mei Sum Woo, Annie Lai King Yip, Chloe Yu Chin, Winnie Pui Yin Tang, Mandy Mun Tse Choy, Katrina Wai Key Tsang, Suzanne C. Ho, Helen Shuk Wah Ma, Samuel Yeung Shan Wong

**Affiliations:** 1 Jockey Club School of Public Health and Primary Care, The Chinese University of Hong Kong, Shatin, Hong Kong, China; 2 CUHK Jockey Club Centre for Osteoporosis Care and Control, The Chinese University of Hong Kong, Hong Kong, China; 3 Woo Mei Sum Psychological Practice, Hong Kong, China; 4 School of Nursing, The Hong Kong Polytechnic University, Hong Kong, China; 5 Department of Psychology, The Chinese University of Hong Kong, Hong Kong, China; 6 School of Nursing, The Chinese University of Hong Kong, Hong Kong, China; 7 The Hong Kong Society for Rehabilitation, Hong Kong, China; 8 Hong Kong Center for Mindfulness, Hong Kong, China

## Abstract

Psychological and behavioural interventions may be effective in reducing menopause-related symptoms. This randomized controlled trial aimed to evaluate the effectiveness of Mindfulness-based Stress Reduction (MBSR) in reducing menopause-related symptoms by comparing with an active control group, the menopause education control (MEC). Symptomatic peri-menopausal and post-menopausal women with mild to moderate symptoms were recruited. The primary outcome was overall menopausal symptoms measured by modified Greene Climacteric Scale (GCS). Secondary outcomes include subscales of the GCS perceived stress, mindfulness and health related Quality of Life. All outcome measures were collected at baseline, 2 months (immediately post intervention), 5 and 8 months (3 and 6 months post intervention respectively). Both MBSR (n = 98) and MEC (n = 99) groups reported a reduction in total GCS score at 8 months. Between group analysis show significant symptom score reduction in MBSR group on Anxiety and Depression subscales of GCS. No differences were found between groups on other GCS subscales and majority of the secondary outcome measures. The findings show that menopausal symptoms in both MBSR and MEC significantly reduced over the study period. MBSR show a greater reduction of psychological symptoms of depression and anxiety above active controls but do not reduce other somatic, urogenital and vasomotor symptoms.

## Introduction

The menopause transition can cause psychological and somatic disturbances in a woman’s life, and over 75% of women may experience some if not all of menopause related symptoms which may include vasomotor symptoms, sleep difficulties, depression and anxiety and urogenital symptoms and sexual dysfunction^1–4^. Meanwhile, negative emotions such as depression and anxiety symptoms and increased social and psychological stress were associated with increased reporting of physical symptoms in menopausal women. It has been suggested that women’s psychological reactions to vasomotor and somatic symptoms may exacerbate these physical symptoms^5–8^, and the National Institutes of Health convened panels recognized the need to evaluate psychological and behavioural interventions which can potentially mediate the reaction to and increase resilience and coping of menopause-related symptoms^9^.

Recent studies of psychological interventions to improve coping such as cognitive behavioral strategies and relaxation techniques have suggested positive effects on vasomotor and depression symptoms. However, studies have been limited by a lack of well-designed randomized controlled studies with adequate sample size^10–15^. Mindfulness-based therapies offer an alternative approach to increase self-acceptance and coping by providing systematic training in mindfulness meditation as a self-learned approach to stress reduction^16^. The Mindfulness-based stress reduction (MBSR) is a clinical program which utilizes skills such as sitting and walking meditation, yoga and a somatically focused skill named the body scan^17–19^. In peri- and post-menopausal women, MBSR trains women to recognize and discriminate accurately the various components of daily life experiences such as thoughts, feelings, and sensations while at the same time, developing a nonreactive awareness and acceptance towards these thoughts, feelings and sensations^20,21^.

Mindfulness based therapies has been effective in improving physical and psychological outcomes in a variety of clinical and non-clinical settings in the last three decades^22–25^, although few studies focus on women with menopausal symptoms^26^.

The first pilot study of MBSR was conducted in 2006 and 15 women with moderate or severe hot flushes using MBSR showed a reduction in the severity of hot flushes and significant changes on the psychosocial scale^8^. However this study lacked a control group. A further study by Carmody *et al*. randomized 110 women with moderate or severe hot flushes to MBSR or as a wait-list control^27^. Results showed a reduction in feeling ‘bothered’ by hot flushes, improved sleep quality, decreased anxiety and perceived stress and improved quality of life. Interestingly, there were no reductions in the severity or frequency of hot flushes. This suggests that reducing negative emotions such as anxiety and stress may help women cope in the menopausal transition. However, due to the lack of an active control group for comparison, it was difficult to delineate the independent effects of MBSR from the non-specific effects of group interactions and additional attention received from instructor and group members.

## Aims and Objectives

The aim of the study was to evaluate the effectiveness of MBSR in reducing menopause-related vasomotor, psychological and somatic symptoms among peri-menopausal and post-menopausal women by comparing the intervention a menopause education control (MEC) group.

We hypothesized that patients randomized to the MBSR group would show a reduction both of menopause-related psychological and somatic symptoms when compared to active controls (MEC).

## Methods

### Study Design

This was a randomized controlled trial with two study arms: (1) an MBSR program led by a trained instructor; and (2) a menopause-education control group. Both MBSR and MEC lasted for eight weeks and the outcome measures were collected at similar time points baseline, 2 months (immediately post intervention), 5 months and 8 months (3 months and 6 months post intervention respectively). The MEC intervention controls for nonspecific factors including instructor attention, group support and physical activity. The MBSR and MEC intervention were both conducted in groups with matching group size and instructor attention of 2.5 hr-session weekly for 8 weeks. Physical activity/techniques consisted of at least 60 minutes. For MEC group, simple stretching exercises were taught for better posture and aiming to improve joint mobility; whilst for MBSR group, mindful exercises were introduced to cultivate nonjudgmental awareness of physical sensations. In both groups, participants were asked to complete the same amount (40 minutes) of home practice.

### Participants

Patients were recruited from community and primary care settings: (1) advertisements invited women between the ages of 40–60 with menopausal symptoms were posted in local newspaper columns; (2) internal emails were sent to the Chinese University of Hong Kong (CUHK) staff, students and alumni; (3) recruitment articles were posted in newsletters of Centre of Research and Promotion of Women’s Health (CRPWH) and Women’s clubs of the Family Planning Association of Hong Kong; (4) posters and leaflets were also distributed in General Outpatient and Family Medicine Clinics of the New Territories East Cluster of the Hospital Authority and the Prince of Wales Centre of the Hong Kong Society for Rehabilitation. The invitation described briefly an 8-week program which involved mindfulness or psychoeducation.

All recruited participants fulfilled the following inclusion criteria: (1) Chinese women aged 40–60 years, who were peri-menopausal or post-menopausal^1,6^. Peri-menopausal status was defined as the period with changes in menstrual pattern and irregular cycle with changes in frequency compared with 12 months ago. Post-menopausal status was defined as absence of periods for at least 12 months; (2) women who were willing to maintain their present exercise and dietary pattern; and (3) women having a somatic symptom score of six or above on the Greene Climacteric Symptom Score. The average complaint on somatic subscale was reported to be 6.16 (SD 4.25) based on 200 Scottish women aged 40–55 years from menopause clinics^28^. Since somatic symptoms have been found to be more common among Hong Kong Chinese women as compared to Caucasians^6^, and local studies showing with a mean score of 5.4–6.4, the score of six on somatic subscale was chosen as one of the inclusion criteria^29,30^. Interested participants were excluded if they: (1) had psychiatric and medical comorbidities that were potentially life-threatening (i.e., psychosis, suicidal ideation, terminal medical illness) or conditions expected to severely limit patient participation or adherence (e.g., psychosis, current substance or alcohol abuse, dementia, and currently pregnant); or (2) had medical conditions or medications that might affect menopausal symptoms including hot flushes (such as the presence of thyroid disease or the use of selective estrogen receptor modulator, hormone therapy, or the use of traditional Chinese medicine for the menopause); (3) women who had previously practiced or were currently practicing meditation. Participants who were using any anxiolytic or antidepressants were asked to keep their present dosage stable and to immediately report any change of medication during the study period; and (4) had hysterectomy or oophorectomy.

All interested subjects were initially screened over the phone by our trained research staff to determine their eligibility. Those who were eligible, individual face-to-face interviews were scheduled to further confirm the inclusion and exclusion criteria by the clinician investigator. The objectives of the study were further explained and informed consent was obtained. All interviews were conducted at the Chinese University of Hong Kong. Participants were randomly assigned to one of the treatment groups by using a list of computer-generated random numbers. The generation of random numbers and assignment was performed by a statistician who was not part of the research team to ensure concealment of randomization. The allocation to the two groups was 1:1. Recruitment stopped when the target sample size was reached. Between Feb 2014 and Jan 2015, six batches of study intervention and active control groups were conducted. Group sizes ranged from 11 to 21. Participants were blinded to the study hypotheses. Ethical approval for the study was granted by the Joint Chinese University of Hong Kong – New Territories East Cluster Clinical Research Ethics Committee (approval number: CRE-2011.561), and procedures were carried out in accordance with the approved guidelines.

### Interventions

#### Mindfulness-based Stress Reduction (MBSR)

The MBSR program utilized stress-reduction skills which included sitting and walking meditation, yoga and body scan^17–19^. The participants were instructed to maintain attention on their immediate experience with an attitude of openness, acceptance, and compassion^16–18,27^. Three instructors (two clinical psychologists and one nurse) were employed to deliver the MBSR programme. All three instructors had received trainings at the UMass Center for Mindfulness, attended retreats and have more than 2 years of teaching experience in MBSR. The recruitment of three instructors was to increase the generalizability (external validity) of the findings and to show that it is not the therapist per se but the therapeutic modality that accounted for changes in outcomes. The Chinese MBSR program followed the standard MBSR protocol, only that it was conducted in Chinese^17^. The Chinese MBSR program has been successfully applied in other studies^31,32^. It consisted of two-and-a-half-hour weekly sessions for eight weeks with a total of 20 hours. The program included 40-minute daily homework exercises that consisted of guided (audiotaped) or unguided awareness exercises directed at increasing moment-by-moment non-judgmental awareness of bodily sensations, thoughts and feelings, together with exercises designed to integrate the application of awareness skills into daily life. The curriculum included training in mindfulness through (1) a body scan, the gradual moving of attention through the body from head to feet while lying on a mat on the floor, bringing awareness particularly to bodily sensations; (2) sitting meditation, in which attention is brought to breathing sensations and the flow of bodily sensations, thoughts, and emotions; and (3) mindful stretching exercises, to cultivate awareness during simple stretching movement^17^. The key themes of MBSR included the empowerment of participants and a focus on awareness and acceptance of experience of the present moment. Participants were guided to develop a “decentered” perspective on thoughts and feelings, in which these were viewed as passing events in the mind. The study MBSR protocol utilised a general approach similar to previous studies^8,27^.

#### Menopause Education Control (MEC)

Psychoeducational programs deliver health information on the menopausal transition in combination with education and exercise in small support groups. This can help women process their experiences on cognitive, emotional and social levels and empower self-care by providing emotional and practical help^33–36^.

The purpose of the MEC was to act as an active control group to match non-specific effects including instructor’s attention and social support from group. The MEC also consisted of two-and-a-half-hour weekly sessions for eight weeks with a total of 20 hours and were conducted according to a protocol used previously in similar research^31^. These sessions included health information about the menopause, self-help and medical treatments, discussion of expectations and beliefs about the menopause and general health advice and discussion (stress reduction, exercise, smoking and diet)^35,37^. Three nurses were employed to lead these sessions and all have at least two year clinical experience in conducting psychoeducation sessions. Each sessions were supplemented with discussion with respect to the content of the educational presentation as well as mutual sharing. Active control group participants were taught simple stretching techniques which focused on physical techniques only and did not require the level of attention and awareness mindfulness practices require. Participants were encouraged to practice stretches for 40-minute daily. Both MBSR and MEC sessions were audiotaped.

### Measures

Subjects’ demographic data, including age, sex, educational status, household income and size, the use of medication (including psychotropics), use of dietary supplements and medical history (including medical and psychiatric history), were collected before their individual interview with the clinician investigator.

#### Primary outcome measures

Greene Climacteric Scale (GCS): the climacteric symptoms were assessed by the Chinese version of the GCS^38,39^. The GCS consists of 21 items that are grouped into five different domains: anxiety (6 items); depression (5 items); somatic symptoms (7 items); vasomotor symptoms (2 items); and sexual symptoms (1 item). The modified GCS was used in this study with the addition of urogenital symptoms with three items: increased urinary frequency, urinary incontinence, and vaginal dryness which was used previously and was validated^38,39^. Each symptom was scored on a four-point rating scale from 0 (not at all bothered) to 3 (extremely bothered), and the total score showed the overall severity of the climacteric symptoms. The instrument was found to be valid and culturally equivalent for clinical assessment of menopausal symptoms in Hong Kong Chinese^39^. The primary outcome measure was the total GCS scores at 8 months (6 months post intervention).

#### Secondary outcome measures

Perceived Stress: Stress was measured by the Global Measure of Perceived Stress Scale (PSS)^40^. The psychometric data for the Global Measure of Perceived Stress revealed robust reliability, with Cronbach alpha coefficients ranging from 0.84–0.86. The Chinese version was used in this study^41^. Internal consistency for the Chinese version of the PSS ranged from 0.79 to 0.85 with test–retest reliability of 0.81.

Health related Quality of Life: Health related quality of life was measured by the validated Chinese version Medical Outcomes Study Short-Form Health Survey (SF-12)^42^. The SF-12 is a 12-item survey that reports health-related quality of life, including both physical (PCS) and mental (MCS) functioning and well-being. It has shown to be a valid measure for measuring health related quality of life in local Chinese population.

Mindfulness: Mindfulness was measured by the Five Facet Mindfulness Questionnaire (FFMQ) to evaluate whether the increase or changes in mindfulness were related to changes in the outcomes of menopausal symptoms^43^. This scale was translated in Hong Kong and had undergone an initial validation process^44^.

Participants were assessed at four timepoints: baseline, 2 months after randomization (immediately post intervention), and at 5 and 8 months (3 and 6 months after intervention respectively) using self-administrated questionnaire including both primary and secondary outcomes. The baseline questionnaires were distributed to participants after their interview with the clinician investigator. Participants did not know the randomization result when completing the baseline questionnaires. The following three questionnaires together with prepaid envelopes were posted by mail. Participants who dropped out during the study period were encouraged to complete the final questionnaire as their last follow-up. If the questionnaire was not returned within 2 weeks, a reminder telephone call was made. A maximum of three follow up calls were made.

### Data and statistical analysis

Baseline characteristics including age, education background, occupation, marital status, religious belief, monthly household incomes and the stages of menopause of the two groups were compared using the independent samples t-test for continuous variables and chi-square test for categorical variables. The primary outcome, the total GCS score at 8 months (6 months after intervention), was analysed using ANCOVA with adjustment of the baseline total GCS scores. Intention to treat (ITT) and per protocol (PP) analyses were conducted using observed and imputed data. ITT population included all participants randomized and PP population included participants received at least six or more sessions. Multiple imputations were used to handle missing data and were done by using the R package mice, version 2.2.5 to create 20 complete datasets by chained equations^45^. Robin’s rule was adopted to calculate the effect estimates from the 20 completed datasets^46^. Secondary analyses included analyses on all subscales of GCS, PSS, FFMQ, and SF-12 at 8 months (6 months post intervention) using ANCOVA with adjustment of the baseline scores. In addition, statistical analysis of changes of primary and secondary outcomes including GCS, PSS, FFMQ and SF-12 overtime were made using linear mixed models. In our models, intervention group, time and the interactions between the intervention group and time were treated as fixed factors, and an unstructured covariance structure was employed. A two-sided p-value of 0.05 or less was considered statistically significant.

### Sample Size

A previous study that evaluated the effects of MBSR on reducing hot flush bother and menopausal related quality of life were used for sample size calculation^27^. Using pilot data that evaluated an existing menopausal education support group run by CRPWH, we assumed similar effect size of MEC of −0.286 and the average R-square value between the response and the covariates of 0.15, with 2 sided type I error of 5% and 85% power to detect statistical significant mean differences between MBSR group and MEC group, the required sample size was 78 participants in each group. Assuming a dropout rate of around 20%, our enrolment target was196 with 98 participants in each group.

### Data Availability

The datasets generated during and/or analysed during the current study are available from the corresponding author upon request.

## Results

**Figure 1:**
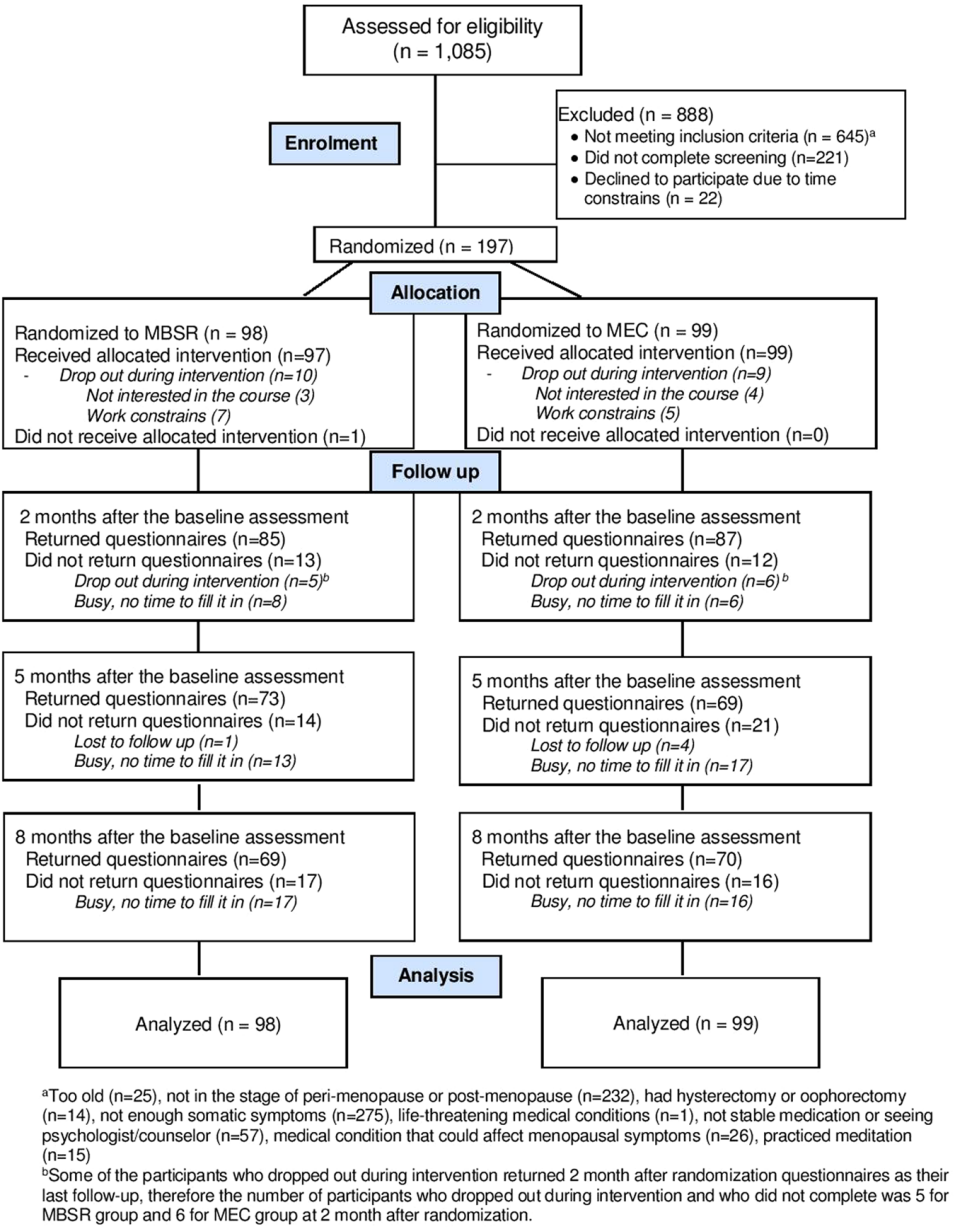
The CONSORT diagram.

Participants were recruited from August 2013 to November 2014. A total of 1085 women were assessed for eligibility, of which 197 participants enrolled in this study, reaching the targeted number for recruitment for this study (Fig. 1). The most common reason for exclusion from the study was under threshold GCS score (n = 275) and women presenting outside the defined peri-menopause or who’s period has stopped for more than 3 years (n = 232). The average age was 52.0 ± 3.09 years old and 60.9% (n = 120) participants were in the peri-menopausal stage during the intervention. At baseline, 5 participants in the MBSR group and 6 participants in the MEC group were on stable psychiatric medication for more than 3 months. Demographics of all participants and the characteristics of the two study arms are presented in Table 1. There were no significant differences between the two groups on demographic variables or the baseline outcome measures, except for the vasomotor subscale of the GCS. No known harms or unintended effects were reported from either of the groups. Due to scheduling conflicts, the three MBSR instructors lead 3, 2, and 1 group(s) respectively. The teaching configuration was mirrored by MEC instructors in teaching 3, 2 and 1 group(s) respectively. Fidelity checks of both groups were conducted by randomly reviewing 10% of the total sessions. The treatment fidelity ratings were 86.17% for MBSR group (82%, 92% and 84.5% respectively) and 95.67% for MEC group (89%, 98% and 100%).

**Table 1:** Demographic characteristics of participants at baseline (n = 197).

Items	Total (n = 197)	MBSR (n = 98)	MEC (n = 99)	*p* value*
**Demographic characteristics**				
Age, mean (sd)	52.0 (3.09)	51.9 (3.0)	52.1 (3.2)	0.323
Education, n (%)				0.115
Primary school or below	11 (5.6)	3 (3.1)	8 (8.1)	
Secondary school	136 (69.0)	69 (70.4)	69 (67.7)	
Diploma	32 (16.2)	19 (19.4)	13 (13.1)	
University or above	18 (9.1)	7 (7.1)	11 (11.1)	
Occupation, n (%)				0.462
Housewife	91 (46.2)	46 (46.9)	45 (45.5)	
Employed	75 (38.1)	36 (36.7)	39 (39.4)	
Retired or unemployed	31 (15.7)	16 (16.3)	15 (15.2)	
Marital Status, n (%)				0.241
Single	20 (10.2)	10 (10.2)	10 (10.1)	
Married	149 (75.6)	72 (73.5)	77 (77.8)	
Separated/Divorced	22 (11.2)	14 (14.3)	8 (8.1)	
Alone/Bereaved	6 (3.0)	2 (2.0)	4 (4.0)	
Religion, n (%)				0.226
None	117 (59.4)	60 (61.2)	57 (57.6)	
Buddhist/Taoist	25 (12.7)	12 (12.2)	13 (13.1)	
Christianity/Catholicism	50 (25.4)	22 (22.4)	28 (28.3)	
Others	5 (2.5)	4 (4.1)	1 (1.0)	
Family Size, mean (sd)	3.1 (1.1)	3.1 (1.1)	3.2 (1.1)	0.187
Monthly Household Income				0.124
$10000 or below	57 (28.9)	26 (26.5)	31 (31.3)	
$10001–$20000	74 (37.6)	38 (38.8)	36 (36.4)	
$20001–$30000	42 (21.3)	25 (25.5)	17 (17.2)	
$30001 or above	23 (11.7)	8 (8.2)	15 (15.2)	
Stage of menopause				0.447
Peri-menopause	120 (60.9)	59 (60.2)	61 (61.6)	
Post-menopause	77 (39.1)	39 (39.8)	38 (38.4)	

^*^p value was based on independent sample t-test for continuous variables and chi-square test for categorical variables.

### Primary Outcome

A reduction of total GCS scores at 8 months (6 months post intervention) was seen in both MBSR and MEC groups. After controlling for participants’ baseline GCS scores, there was a significant effect of group membership on participants’ total GCS scores at 8 months. A significant reduction of total GCS scores was seen in the MBSR group on total GCS score compared to MEC group. The mean difference between MBSR group and MEC group is −3.27 (−5.51 to −1.03, p = 0.005, Cohen’s d = −0.49) for ITT population using observed data only (Table 2). The results were the same for PP population as well as when imputed data was used.

**Table 2:** ITT result on primary and secondary outcomes based on observed data.

	MBSR^a^	MEC^a^	Between group difference^*b*^	*p* value	Cohen’s *d*
**Primary outcome**					
*GCS total score*					
Baseline	31.72 (10.23); 98	30.68 (8.35); 99			
8 months	23.52 (8.93); 69	26.04 (8.84); 70	−3.27 (−5.51 to −1.03)	**0.005**	0.49
**Secondary outcome**					
*GCS-Anxiety*					
Baseline	8.23 (2.96); 98	8.00 (2.48); 99			
8 months	5.97 (2.49); 69	6.97 (2.57); 70	−0.99 (−1.71 to −0.28)	**0.007** ^***c***^	0.46
*GCS-Depression*					
Baseline	6.96 (2.95); 98	6.59 (2.45); 99			
8 months	4.78 (2.60); 69	5.39 (2.41); 70	−0.77 (−1.47 to −0.07)	**0.031**	0.24
*GCS-Somatic*					
Baseline	8.79 (3.09); 98	9.03 (2.98); 99			
8 months	6.39 (2.94); 69	7.44 (3.52); 70	−0.80 (−1.66 to 0.07)	0.072	0.31
*GCS-Vasomotor*					
Baseline	2.78 (1.55); 98	2.29 (1.59); 70			
8 months	2.28 (1.73); 69	2.10 (1.36); 70	−0.28 (−0.69 to 0.13)	0.185	0.23
*GCS-Urogenital*					
Baseline	3.50 (1.91); 98	3.44 (1.80); 99			
8 months	2.84 (1.76) 69	2.89 (1.55) 70	−0.19 (−0.61 to 0.25)	0.397	0.14
*GCS-Sexual*					
Baseline	1.47 (0.94); 98	1.32 (0.79); 99			
8 months	1.26 (1.00); 69	1.26 (0.81); 70	−0.15 (−0.41 to 0.11)	0.248	0.20
*PSS*					
Baseline	28.98 (7.11); 98	28.15 (5.32); 99			
8 months	25.33 (7.65); 69	26.96 (6.89); 70	−1.70 (−3.59 to 0.18)	0.076	0.30
*FFMQ-Observe*					
Baseline	22.00 (5.26); 98	22.48 (5.25); 99			
8 months	23.86 (4.82); 69	22.54 (5.17); 70	1.74 (0.33 to 3.16)	**0.016**	0.59
*FFMQ-Describe*					
Baseline	24.50 (5.44); 98	24.53 (5.25); 99			
8 months	25.51 (4.50); 69	24.94 (4.64); 70	0.19 (−1.04 to 1.26)	0.851	0.12
*FFMQ-Act with Awareness*					
Baseline	24.19 (5.33); 98	26.16 (5.00); 99			
8 months	25.16 (4.87); 69	26.03 (5.05); 70	0.03 (−1.22 to 1.28)	0.962	0.12
*FFMQ-Nonjudge*					
Baseline	23.84 (4.10); 98	23.28 (4.45); 99			
8 months	23.35 (3.92); 69	24.20 (4.10); 70	−0.81 (−1.98 to 0.36)	0.172	0.18
*FFMQ-Nonreact*					
Baseline	20.13 (3.45); 98	19.98 (3.12); 99			
8 months	21.03 (3.33); 69	20.27 (3.79); 70	0.72 (−0.23 to 1.67)	0.135	0.22
*PCS*					
Baseline	38.51 (7.50); 98	38.58 (8.64); 99			
8 months	41.83 (7.73); 69	41.54 (7.85); 70	0.73 (−1.48 to 2.93)	0.517	−0.11
*MCS*					
Baseline	39.66 (10.03); 98	39.67 (8.95); 99			
8 months	43.53 (9.86); 69	42.52 (9.83); 70	0.76 (−2.01 to 3.54)	0.587	−0.10

Primary and secondary outcomes at 8 months.

^a^Mean (SD); n ^b^Mean (95% CI) ^c^Different result reported on ITT analysis using both observed and imputed data (*p* = 0.070).

MBSR – Mindfulness Based Stress Reduction; MEC - Menopause Psycho-education control; GCS - Greene Climacteric Scale; PSS – Perceived Stress Scale; FFMQ – Five Facet Mindfulness Questionnaire; PCS – SF12 physical component; MCS – SF12 mental component; ITT- Intention to treat.

### Secondary Outcome

To look at the group effect on subscales of GCS, ANCOVA analyses controlling for baseline scores using observed data revealed significant reduction of MBSR on anxiety and depression subscales, but not on somatic, vasomotor, sexual and urogenital subscales were used. Group effects on anxiety (F(1,35) = 7.53, *p* = 0.007) and depression (F(1,21) = 4.78, p = 0.031) were significantly different for ITT population using observed data only. The results were similar, except on anxiety subscale using both observed and imputed data for ITT population (p = 0.070). No significant differences were reported between MBSR and MEC on secondary outcome measures which included PSS, FFMQ and SF-12 (mental and physical components).

**Figure 2:**
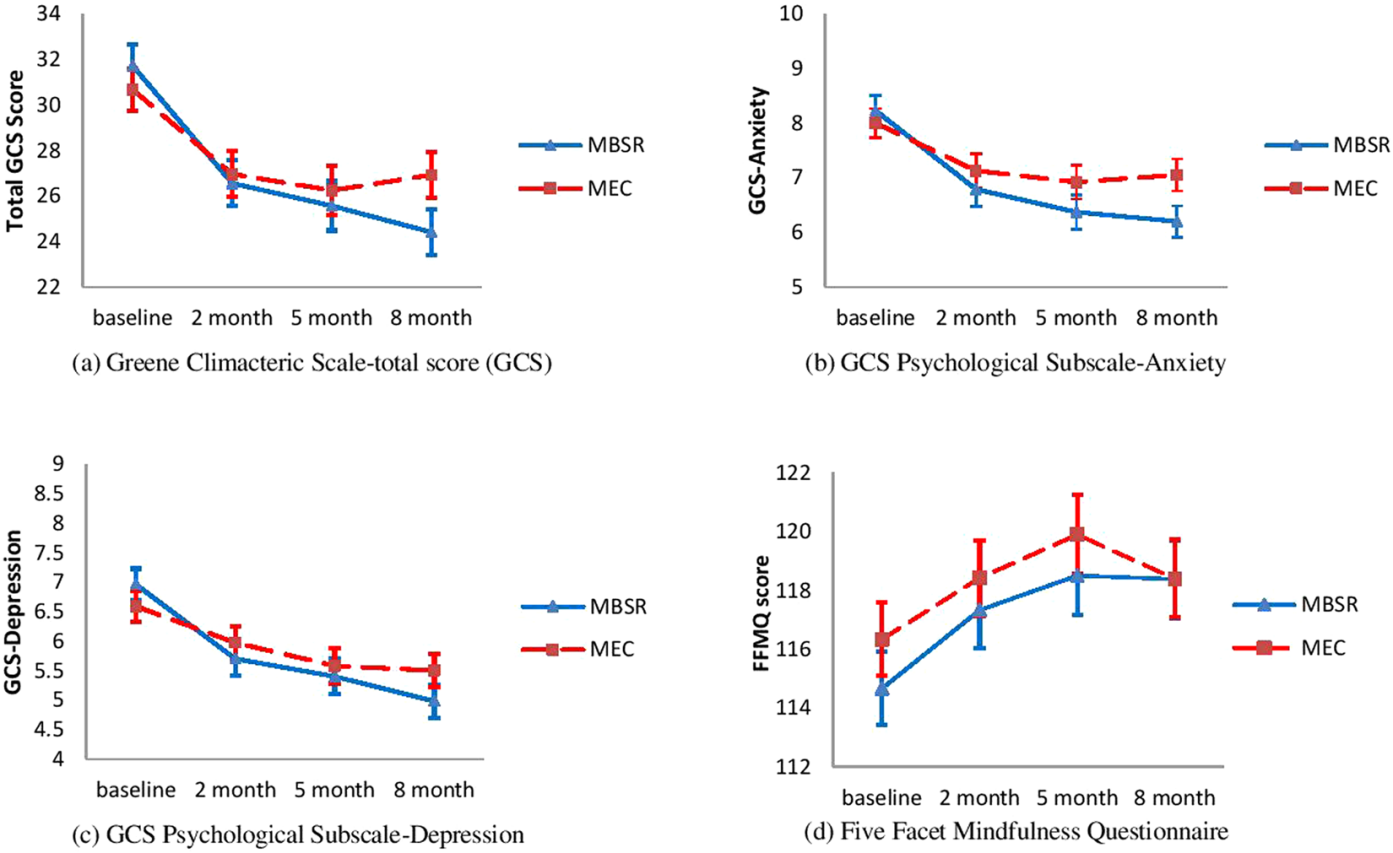
Estimated mean scores for patients reported outcomes in MBSR and MEC group over the study period. Error bars represent the 95% confidence intervals.

Secondary analyses using data collected at all time points including baseline, 2 months, 5 months and 8 months were conducted using Linear Mixed Model (LMM) following the intention to treat principle on all outcome measures. The LMM results on total GCS scores indicated significant Time effect (F(3,159) = 38.63, p < 0.0001) and Group x Time interaction (F(3,159) = 3.41, p = 0.019), suggesting that menopausal symptoms improved over the study period and the group effect varied with time. The estimated means and 95% confidence intervals generated by the LMM procedure were presented in the trajectories in Fig. 2(a). Significant Time effects were reported on all sub scales except for sexual dysfunction. Improvements were reported on anxiety, depression, somatic, vasomotor and urogenital subscales in both MBSR and MEC groups. Significant main effect of Time was also reported on PSS and SF-12, indicating that improvements took place for all participants regardless of their group allocation. However, no Group × Time interaction were seen in any of the secondary outcome measures, suggesting the improvements observed in the two groups do not significantly differ from each other.

### Treatment Adherence

On average, the MBSR group attended 6.29 ± 2.03 sessions, whereas the MEC group attended 6.08 ± 1.98 session. There was no significant difference between the two groups on the number of sessions attended. Seventy-two (73.5%) participants who received MBSR and 70 (70.7%) participants who received MEC attended ≥6 sessions. The main reason for absence was due to participants’ unexpected scheduling conflict. Ten participants randomized to MBSR group and nine participants randomized to MEC group dropped out during intervention, the reasons were 1) not interested in the course (n = 7), 2) work constraints (n = 12). At 5 months after baseline assessment, 41 (41.8%) participants in MBSR group and 39 (39.41%) participants in the MEC group reported that they still practiced the techniques taught during class. The number of participants who kept practice was 33 (33.7%) in the MBSR group and 28 (28.3%) in the MEC group at 8 months after baseline assessment. There is no significant relationship between practice and study outcomes.

## Discussion

To our knowledge, this is the largest reported study of MBSR for menopausal symptoms (n = 197) and the first in comparing MBSR with active controls using a psychoeducation group (MEC). This study aimed to seek the independent effects of mindfulness above non-specific effects such as small group support and instructor’s attention. Both MBSR and MEC were shown to reduce overall menopausal symptoms using the Greene Climatic Scale (GCS) over time. There appears to be a significant reduction of anxiety and depression symptoms in the MBSR group.

Consistent with the study by Carmody *et al*.^27^, we have demonstrated positive MBSR effects on anxiety, although we were unable to replicate positive effects on vasomotor symptoms and perceived stress over active controls. Our study did not particularly target women with moderate or severe hot flushes or night sweats as the inclusion criteria focus on symptoms commonly exhibited in a Chinese population^47,48^. The women in our study were, slightly younger (with mean age of 52.0 years than 53.1 years)^27^, of a lower educational level (postgraduate 9.1% vs. 38%)^27^ and likely to have mild to moderate vasomotor symptoms.

Previous studies of behavioural therapies on menopausal symptoms reporting positive outcomes involve various types of relaxation or respiration techniques from progressive muscle relaxation^49^, paced respiration^50,51^ or applied relaxation techniques^52,53^. However, these studies were limited by small sample sizes (all n < 35). Physical exercise with meditation or relaxation i.e. yoga had shown some improvement in psychological symptoms and musculoskeletal pain when compared with exercise or walking; studies were small or had methodological limitations^54–57^. In this study, active controls (MEC) had elements of physical exercise using stretching but did not involve meditation, relaxation or respiration techniques of MBSR. The positive effects of MBSR on psychological symptoms of anxiety and depression have previously been extensively documented and is consistent with our study findings^58–60^. Despite positive effects of MBSR on psychological symptoms, we have demonstrated no significant reduction of somatic and vasomotor symptoms above active controls (MEC). The study suggests that components of MBSR e.g. acceptance, meditation, relaxation and paced respiration have little effect on the perception and coping of other menopause-related symptoms.

Despite the reduction of menopausal psychological symptoms in the MBSR group, we were unable to demonstrate a difference of mindfulness levels between MBSR and MEC groups, except on the Observe subscale, to explain the positive effects of MBSR on menopausal symptoms. Mindfulness levels (FFMQ) increased in both the MBSR and MEC groups. This phenomenon has also been demonstrated in other studies of mindfulness and active controls which used self-reported questionnaire to measure the levels of mindfulness^33^. The improvement of overall menopausal symptoms and psychological symptoms in MBSR may be facilitated by other mediatory mechanisms such as relaxation, paced respiration or other unmeasured complex psychological pathways e.g. increase in acceptance. An alternative explanation is the complexity and difficulty in assessing outcomes in mindfulness levels and/or other mechanisms. Some have also argued that mindfulness is a difficult construct to be measured by questionnaire and there is a need to improve the existing scales with higher discriminant ability^34^. MEC components may also elicit constructs of MBSR in acceptance and psychological processes through small group support, information giving and physical exercise. Menopausal symptoms declined in both MBSR and MEC groups in our study. Numerous studies on psychoeducation programs have show positive effects from baseline symptoms, although none utilised an RCT approach to compare with usual care or no treatment^35–37,61,62^. Further studies are needed to differentiate the mechanism of effect given that there are psychological, behavioural, group and attention components to MBSR and MEC groups and to compare with the natural course of symptoms in menopause.

The strength of our study includes the ability to evaluate overall menopausal symptoms using a multidimensional tool (GCS) to differentiate anxiety and depression symptoms and to encompass the spectrum of menopausal symptoms. Our study is the largest trial for psychological therapies for menopausal symptoms and is the first to encompass an active control group, with the longest follow up of 6 months post intervention. In addition, we included both peri-menopausal and post- menopausal women with non-selective approach to menopausal symptoms.

### Limitations

The women in our study may not be representative of the general female population with menopausal symptoms within the region of Hong Kong. Those in full time employment constitute 38.1% of our study population whilst full time employment was 63% for women aged between 50–54 years^63^. Self- selection bias is likely as the program require regular attendance and time commitment of participants. Characteristics of self-selection bias e.g. social support, loneliness may be further explored in further studies. Meanwhile, those attending are likely representative of those most willing to attend should such programs become available.

Although a clinician investigator excluded those who had life threatening psychiatric illness and assessed participants as being stable on current medication for depression and anxiety, no validated screening tools were used to assess psychological symptoms apart from the GCS depression and anxiety subscales. In addition, there has been a lack of studies in comparing GCS anxiety and depression subscales with validated scales of anxiety and depression. A cut-off score of 10 had been recommended for clinically anxious or depression according to a study comparing the GCS and the Hospital Anxiety and Depression Scale^28^. Using this cut off, there were no significant differences of clinically relevant anxiety and depression symptoms between MBSR and MEC groups. The mean score was presented in Table 2.

In addition, the time effects in reduction of anxiety, depression, somatic, vasomotor and urogenital subscales may in part be due to the natural course of menopause rather than the interventions. This is difficult to assess, as there was no inactive control group in our study.

Meanwhile, the research intervention was delivered by experienced personnel (MEC) and may require highly trained personnel (MBSR), which may limit the feasibility of these interventions in the community.

## Conclusion

This large randomized control trial of MBSR and active controls (MEC) in symptomatic peri-menopausal and postmenopausal women show that menopausal symptoms in both MBSR and MEC significantly reduced over time of the study period of 8 months (6 months post intervention). MBSR show a greater reduction of psychological symptoms of depression and anxiety above active controls but similar reduction in somatic, urogenital and vasomotor symptoms.

### Trial Registration

Centre for Clinical Trials, Clinical Trials Registry – Chinese University of Hong Kong, trial number: CUHK_CCT00365 (https://www2.ccrb.cuhk.edu.hk/registry/public/204) and Chinese Clinical Trials Registry, trial number ChiCTR-TRC-13003192 (http://www.chictr.org.cn/showprojen.aspx?proj=6367).

## References

[1] Ho SC (1999). Menopausal symptoms and symptom clustering in Chinese women. Maturitas..

[2] Chan SG, Ho SC, Yip YB, Cheng YK (1997). Health status and climacteric complaints in perimenopausal women in Hong Kong. Maturitas (Supplement).

[3] Huang KE, Xu L, Nasri N, Jaisamrarn U (2010). The Asian Menopause Survey: Knowledge, perceptions, hormone treatment and sexual function. Maturitas.

[4] Avis NE, Crawford SL, McKinlay SM (1997). Psychosocial, behavioural, and health factors related to menopause symptomatology. Women’s Health.

[5] Reynolds F (2000). Relationships between catastrophic thoughts, perceived control and distress during menopausal hot flushes: exploring the correlates of a questionnaire measure. Maturitas.

[6] Yang D (2008). Menopausal symptoms in mid-life women in southern China. Climacteric..

[7] Toral VM (2013). Psychosocial interventions in perimenopausal and postmenopausal women: A systematic review of randomised and non randomised trials and non controlled studies. Maturitas..

[8] Carmody JF, Crawford S, Churchill L (2006). A pilot study of mindfulness-based stress reduction for hot flashes. Menopause..

[9] National Institutes of Health (2005). National Institutes of Health state-of-the-science conference statement: management of menopause-related symptoms. Ann. Intern. Med..

[10] Towey M, Bundy C, Cordingley L (2006). Psychological and social interventions in the menopause. Curr Opin Obstet Gynecol..

[11] Tremblay A, Sheeran L, Aranda S (2008). Psychoeducational interventions to alleviate hot flashes: a systematic review. Menopause..

[12] Butler C, Chapman E, Forman M, Beck AT (2006). The empirical status of cognitive-behavioural therapy: a review of meta-analyses. Clin Psychol Rev..

[13] Ayers B, Smith M, Hellier J, Mann E, Hunter MS (2012). Effectiveness of group and self-help cognitive behavior therapy in reducing problematic menopausal hot flushes and night sweats (MENOS 2): a randomized controlled trial. Menopause..

[14] Keefer L, Blanchard EB (2005). A behavioral group treatment program for menopausal hot flashes: results of a pilot study. App Psychophysiol Biofeedback..

[15] Hunter MS, Liao KLM (1996). Evaluation of a four-session cognitive-behavioural intervention for menopausal hot flashes. Brit J Health Psychol..

[16] Astin JA (1997). Stress reduction through mindfulness meditation: effects on psychological symptomatology, sense of control and spiritual experiences. Psychother Psychosom..

[17] Kabat-Zinn, J. *Full catastrophe living: using the wisdom of your body and mind to face stress, pain, and illness*. (Delacourt 1990).

[18] Carlson LE, Garland SN (2005). Impact of mindfulness-based stress reduction on sleep, mood, fatigue symptoms in cancer patients. International J. Behav. Medicine..

[19] Shapiro SL, Bootzin RR, Figueredo AJ, Lopez AM, Schwartz GE (2003). The efficacy of mindfulness based stress reduction in the treatment of sleep disturbance in women with breast cancer. An exploratory study. J. Psychosomatic Res..

[20] Carmody J (2009). Evolving conceptions of mindfulness in clinical settings. J. Cogn Psychoter..

[21] Teasdale J (1999). Metacognition, mindfulness and the modification of mood disorders. Clin Psychol Psychother..

[22] Chiesa A, Serretti A (2010). A systematic review of neurobiological and clinical features of mindfulness meditations. Psychol Med..

[23] Ledesma D, Kumano H (2009). Mindfulness based stress reduction and cancer: a meta-analysis. Psychooncology..

[24] Smith JE, Richardson J, Hoffman C, Pilkington K (2005). Mindfulness based stress reduction as supportive therapy in cancer care: systematic review. J. Adv Nurs..

[25] Chiesa A, Serretti A (2009). Mindfulness-based stress reduction for stress management in healthy people: a review and meta-analysis. J. Altern. Compl. Med..

[26] Green SM, Key BL, McCabe RE (2015). Cognitive-behavioral, behavioral, and mindfulness-based therapies for menopausal depression: a review. Maturitas..

[27] Carmody JF (2011). Mindfulness training for coping with hot flashes: results of a randomized trial. Menopause..

[28] Greene, J. G. Guide to the Greene climacteric scale. *Glasgow* 1991.

[29] Lam PM (2004). A randomized, placebo-controlled, crossover study of tibolone (Livial) on menopause symptoms, psychological well-being, and dyadic relationship of postmenopausal Chinese women and their spouses. Menopause..

[30] Lam PM (2004). Psychological well-being and the dyadic relationship of Chinese menopausal women (and their spouses) attending hormone replacement clinics. Gynecol Endocrinol..

[31] Wong SYS (2011). Comparing the effectiveness of Mindfulness-based Stress Reduction and multidisciplinary programs for chronic pain: a randomized comparative trial. Clin J Pain..

[32] Hou J (2013). Mindfulness-based Stress Reduction Program Improves Family Caregivers’ Mental Well-being: A Randomized Controlled Trial. Psychother Psychosom..

[33] Wong SY (2016). Mindfulness-based cognitive therapy v. group psychoeducation for people with generalised anxiety disorder: randomized controlled trial. Br J Psychiatry..

[34] Sauer, S. *et al*. Assessment of Mindfulness: Review on State of the Art. Mindfulness. 4, 3–17 (2013).

[35] Rotem M, Kushnir T, Levine R, Ehrenfeld M (2005). A Psycho-Educational Program for Improving Women’s attitudes and Coping with Menopausal Symptoms. J Obst, Gynaecol Neonatal Nurs..

[36] Boggs PP, Rosenthal MB (2000). Helping women help themselves: developing a menopause discussion group. Clin Obstet Gynaecol..

[37] Hunter, M. O’Dea. An evaluation of a health education intervention for mid-aged women: five year follow up of effects upon knowledge, impact of menopause and health. Patient educ couns. **38**, 249–55 (1999). 10.1016/s0738-3991(98)00143-8 10865690

[38] Greene J (1998). Constructing a standard climacteric scale. Maturitas..

[39] Chen RQ, Davis SR, Wong CM, Lam TH (2010). Validity and cultural equivalence of the standard Greene Climacteric Scale in Hong Kong. Menopause..

[40] Cohen S, Kamarck T, Mernelstein R (1983). A gobal measure of perceived stress. J. Health Soc. Behav..

[41] Chu LC (2005). Chinese PSS (14 Item). Chinese J Psycho..

[42] Lam CLK, Tse EYY, Gandek B (2005). Is the standard SF-12 health survey valid and equivalent for a Chinese population. Qual Life Res..

[43] Baer RA, Smith GT, Hopkins J, Krietemeyer J, Toney L (2006). Using self-report assessment methods to explore facets of mindfulness. Assessment..

[44] Hou J, Wong SYS, Lo HHM, Mak WWS, Ma HSW (2014). Validation of a Chinese Version of the Five Facet Mindfulness Questionnaire in Hong Kong and Development of a Short Form. Assessment..

[45] van Buuren S, Groothuis-Oudshoorn K (2011). Mice: multivariate imputation by chained equations in R. J Stat Softw..

[46] Rubin, D. B. *Multiple imputation for nonresponse in surveys*. (John Wiley & Sons, 2004).

[47] Ho SC, Chan SG, Yip YB, Chan SY, Sham A (2003). Factors associated with menopausal symptom reporting in Chinese midlife women. Maturitas..

[48] Liu J, Eden J (2007). Experiences and attitude toward menopause in Chinese women living in Sydney: A cross sectional survey. Maturitas..

[49] Germaine LM, Freedman RR (1984). Behavioral treatment of menopausal hot flashes: evaluation by objective methods. J Consult Clin Psychol..

[50] Freeman RR, Woodward S, Brown B, Javaid JI, Pandey GN (1995). Biochemical and thermoregulatory effects of behavioural treatment for menopausal hot flashes. Menopause..

[51] Freeman RR, Woodward S (1992). Behavioral treatment of menopausal hot flushes: evaluation by ambulatory monitoring. Am J Obstet Gynecol..

[52] Irvin JH, Domar AD, Clark C, Zuttermeister PC, Friedman R (1996). The effects of relaxation response training on menopausal symptoms. J Psychosom Obstet Gyaecol..

[53] Nedstrand E, Wijma K, Wyon Y, Hammar M (2005). Applied relaxation and oral estradiol treatment of vasomotor symptoms in postmenopausal women. Maturitas..

[54] Chattha R, Nagarathna R, Padmalatha V, Nagendra HR (2008). Effect of yoga on cognitive functions in climacteric syndrome: a randomized control study. BJOG: An International Journal of Obstetrics & Gynaecology..

[55] Chattha R, Raghuram N, Venkatram P, Hongasandra NR (2008). Treating the climacteric symptoms in Indian women with an integrated approach to yoga therapy: a randomized control study. Menopause.

[56] Elavsky S, McAuley E (2007). Lack of perceived sleep improvement after 4-month structured exercise programs. Menopause..

[57] Elavsky S, McAuley E (2007). Physical activity and mental health outcomes during menopause: a randomized controlled trial. Ann Behav Med..

[58] Carmody J, Baer RA (2008). Relationships between mindfulness practice and levels of mindfulness, medical and psychological symptoms and well-being in a mindfulness-based stress reduction program. J Behav Med..

[59] Goyal M (2014). Meditation Programs for psychological stress and well-being: a systematic review and meta-analysis. JAMA Intern Med..

[60] Würtzen H (2013). Mindfulness significantly reduces self-reported levels of anxiety and depression: results of a randomised controlled trial among 336 Danish women treated for stage I-III breast cancer. Eur J Cancer..

[61] Ueda M (2004). A 12 week structured education and exercise program improved climacteric symptoms in middle aged women. J Physiol Anthropol Appl Human Sci..

[62] Liao KL, Hunter MS (1998). Preparation for menopause: prospective evaluation of educational intervention for mid-aged women. Maturitas..

[63] Women’s Commission. Hong Kong women in figures 2015. *figshare* http://www.women.gov.hk/download/research/HK_Women2015_e.pdf (2015).

